# Streptococcus mutans adherence to conventional and self-ligating brackets: an in vitro study

**DOI:** 10.1590/2177-6709.26.6.e212019.oar

**Published:** 2021-12-15

**Authors:** Murilo Fernando Neuppmann FERES, Fernanda VICIONI-MARQUES, Fábio Lourenço ROMANO, Marina Guimarães ROSCOE, Vinícius Matsuzaki de SOUZA, Aline Lira TORNERI, Bruno BUENO-SILVA

**Affiliations:** 1 Universidade de São Paulo, Faculdade de Odontologia de Ribeirão Preto, Departamento de Clínica Infantil (Ribeirão Preto/SP, Brazil); 2 Universidade de São Paulo, Faculdade de Odontologia, Departamento de Biomateriais e Biologia Oral (São Paulo/SP, Brazil); 3 Universidade de Guarulhos, Programa de Pós-Graduação, Mestrado em Ortodontia (Guarulhos/SP, Brazil); 4 Private practice (Guarulhos/SP, Brazil)

**Keywords:** Orthodontic brackets, Biofilms, *In vitro* techniques

## Abstract

**Introduction:**

Although self-ligating brackets presumably provide better hygiene conditions, no consensus has been reached so far.

**Objective:**

Therefore, the objective of this study was to evaluate, in an *in vitro* experimental design, the adherence of *Streptococcus mutans* (SM) in self-ligating and conventional brackets of different manufacturers and ligature types.

**Methods:**

Four commercial brands of maxillary premolar metal brackets were tested (Abzil®; Morelli®; 3M Unitek®; and GAC®). Each one was subdivided into three groups, which varied according to the type of ligature and bracket model (metallic, elastic, and self-ligating), totalizing twelve groups, composed of six brackets each. Previously sterilized brackets were initially immersed in saliva for one hour, and subsequently washed and added in a bacterial suspension, maintained in aerobiosis for 72 hours. The adhered bacteria were then separated and quantified by colony forming units (CFU/mL) counting after 48 hours of growth. The groups were compared by Kruskal-Wallis and Dunn *post-hoc* tests (*p*< 0.05).

**Results:**

Regardless of the commercial brand, self-ligating brackets had significantly less CFU/mL. However, according to comparisons performed within each commercial brand, only Abzil® self-ligating brackets had significantly lower biofilm adhesion. Among all of the self-ligating models, GAC® brackets presented the highest bacterial adhesion rate.

**Conclusions:**

Self-ligating brackets are likely to present lower rates of biofilm adhesion. Particularly, Abzil® and GAC® self-ligating brackets are less likely to accumulate biofilm. Although such results are derived from an *in vitro* study, practitioners might acknowledge findings concerning bacterial adhesion as one of the relevant features to be considered during bracket selection.

## RESUMO

**Introdução:**

Embora os braquetes autoligados presumivelmente proporcionem melhores condições de higiene, nenhum consenso foi alcançado até o momento.

**Objetivo:**

O objetivo do presente estudo foi avaliar, em um delineamento experimental *in vitro*, a aderência de *Streptococcus mutans* em braquetes autoligados e convencionais de diferentes fabricantes e tipos de ligaduras.

**Métodos:**

Quatro marcas comerciais de braquetes metálicos de pré-molares superiores foram testadas (Abzil®; Morelli®; 3M Unitek®; e GAC®). Cada marca foi subdividida em três grupos, que variaram de acordo com o tipo de ligadura e o modelo do braquete (metálico, elástico e autoligado), totalizando doze grupos, compostos por seis braquetes cada. Os braquetes previamente esterilizados foram, inicialmente, imersos em saliva por uma hora, sendo posteriormente lavados e adicionados em uma suspensão bacteriana, mantida em aerobiose por 72 horas. As bactérias aderidas foram, então, separadas e quantificadas por contagem de unidades formadoras de colônias (UFC/mL) após 48 horas de crescimento. Os grupos foram comparados pelos testes *post-hoc* de Kruskal-Wallis e Dunn (*p*< 0,05).

**Resultados:**

Independentemente da marca comercial, os braquetes autoligados apresentaram significativamente menos UFC/mL. Porém, de acordo com as comparações realizadas dentro de cada marca comercial, apenas os modelos autoligados Abzil® apresentaram adesão de biofilme significativamente menor. Entre todos os modelos autoligados, os braquetes GAC® apresentaram a maior taxa de adesão bacteriana.

**Conclusões:**

Os braquetes autoligados provavelmente apresentam menores taxas de adesão do biofilme. Particularmente, os braquetes autoligados Abzil® e GAC® têm menor probabilidade de acumular biofilme. Embora tais resultados sejam derivados de um estudo *in vitro*, os clínicos podem reconhecer os achados relativos à adesão bacteriana como uma das características relevantes a serem consideradas durante a seleção dos braquetes.

## INTRODUCTION

Even though orthodontic treatment brings important positive clinical and psychological effects,^1^ ^-^ ^4^ it is still likely to cause side effects, such as external root resorption and vertical reduction of the alveolar bone crest.^5^ ^-^ ^9^ In addition, orthodontic full-fixed appliances may also complicate oral hygiene,^10^ ^,^ ^11^ resulting in significant biofilm accumulation around the brackets bases.^11^ ^-^ ^14^ As a consequence, this accumulation can lead to negative alterations, such as gingivitis,^15^ ^-^ ^17^ enamel demineralization, including the formation of white spots lesions.^15^ ^,^ ^20^ ^,^ ^21^ Furthermore, orthodontic patients’ installed biofilm profile may also be negatively altered,^22^ ^-^ ^26^ with simultaneous increase and deterioration of the microbiota quality^25^.

One of the most recent advances in Orthodontics refers to the development of the self-ligating brackets, originally designed to facilitate wire insertion and removal.^27^ ^,^ ^28^ These brackets feature an active or passive opening and closing device that ensures a safe and effective engagement of the wire into the bracket slot,^29^ with no need for metallic or elastic ligatures.

Although some authors have claimed that self-ligating brackets provide better hygiene conditions,^29^ ^,^ ^30^ no consensus has been found indicating that self-ligating brackets are actually more advantageous in this aspect.^31^ Therefore, the objective of this study was to evaluate, in an *in vitro* experimental design, the adherence of *Streptococcus mutans* (SM) in self-ligating and conventional brackets of different manufacturers and ligature types.

## MATERIAL AND METHODS

### BRACKETS PREPARATION

Four models of maxillary premolar metal brackets were tested in this study, *i.e.*: Abzil^®^ (São José do Rio Preto, SP, Brazil), Morelli^®^ (Sorocaba, SP, Brazil), 3M Unitek^®^ (Monrovia, CA, USA), and GAC^®^ (Bohemia, NY, USA).

**Figure 1:**
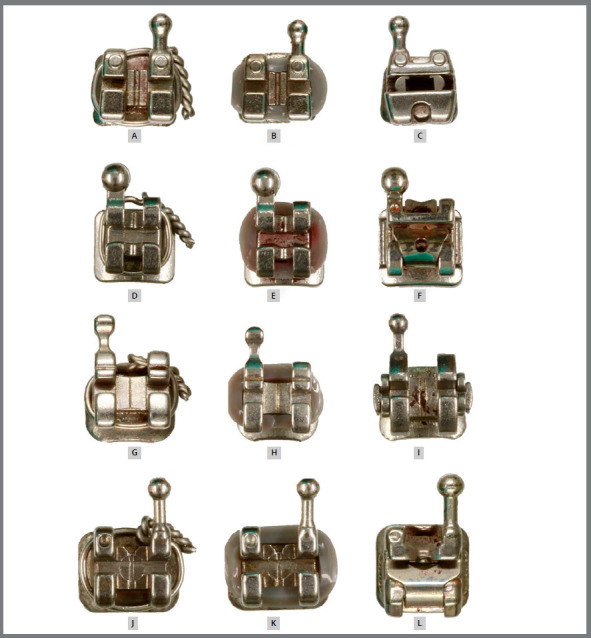
Study groups, in relation to commercial brand and ligature type. Legends: A) Abz-Met; B) Abz-Ela; C) Abz-SL; D) Mor-Met; E) Mor-Ela; F) Mor-SL; G) 3M-Met; H) 3M-Ela; I) 3M-SL; J) GAC-Met; K) GAC-Ela; L) GAC-SL.

For each of these commercial brands, two bracket models were selected (conventional and self-ligating brackets); and two types of ligatures - metallic (0.025 mm; Morelli^®^, Sorocaba/SP, Brazil) or elastic (gray color; Morelli^®^, Sorocaba/SP, Brazil) - were attached to the conventional brackets (Table 1). Thus, a total of twelve groups composed of six brackets each were formed (Fig 1). Each set of brackets was sterilized (Cristófoli^®^, Curitiba/PR, Brazil) at 122ºC for 15 minutes, and then reserved until the experiment.

**Table 1:** Distribution and characterization of study groups in relation to commercial brand, ligature type, nomination and bracket model.

Brand	Ligature	Nomination	n	Model
Abzil^®^	Metallic	Abz-Met	6	Kirium Roth (0.022-in)
	Elastic	Abz-Ela	6	Kirium Roth (0.022-in)
	Self-ligating	Abz-SL	6	Portia Roth (0.022-in)
Morelli^®^	Metallic	Mor-Met	6	Standard Roth (0.022-in)
	Elastic	Mor-Ela	6	Standard Roth (0.022-in)
	Self-ligating	Mor-SL	6	SLI Roth (0.022-in)
3M Unitek^®^	Metallic	3M-Met	6	Victory Series Roth (0.022-in)
	Elastic	3M-Ela	6	Victory Series Roth (0.022-in)
	Self-ligating	3M-SL	6	Smartclip Roth (0.022-in)
GAC^®^	Metallic	GAC-Met	6	Ovation Roth (0.022-in)
	Elastic	GAC-Ela	6	Ovation Roth (0.022-in)
	Self-ligating	GAC-SL	6	In-Ovation Roth (0.022-in)

### SALIVA COLLECTION

Saliva was collected from three voluntary donors and subsequently centrifuged and sterilized by vacuum filtration. The donors were 30 to 36 years old, had good oral health and, at the time of the collection, had fasted for eight hours without having brushed their teeth. In addition, they had not undergone professional cleaning or antibiotic therapy in the three months prior to collection, nor had they had caries or periodontal disease at the time. After collection, the saliva was kept on ice until its use.

### *STREPTOCOCCUS MUTANS* BIOFILM FORMATION 

A *Streptococcus mutans* UA159 strain was initially reactivated from stock cultures in liquid BHI (Brain-heart infusion) medium for 18 to 24 hours at 37ºC, 5% CO_2_, and then cultured in BHI agar plates. After bacterial growth, the individual colonies were removed with the aid of a platinum loop, and then suspended in a solution with liquid BHI medium, to perform the bacterial growth curve. After the *Streptococcus mutans* (SM) culture had reached the LOG phase (OD = 0.5 nm to 660 nm), it was homogenized, and a 100 µL volume of the SM suspension was inoculated into 100 mL BHI medium plus 1% sucrose, in order to obtain a bacterial concentration of approximately 1 to 2 x 10^5^CFU/mL (CLSI, 2012)^32^, which would later be used as an inoculum for biofilm formation in brackets.

### DESCRIPTION OF THE EXPERIMENT

**Figure 2:**
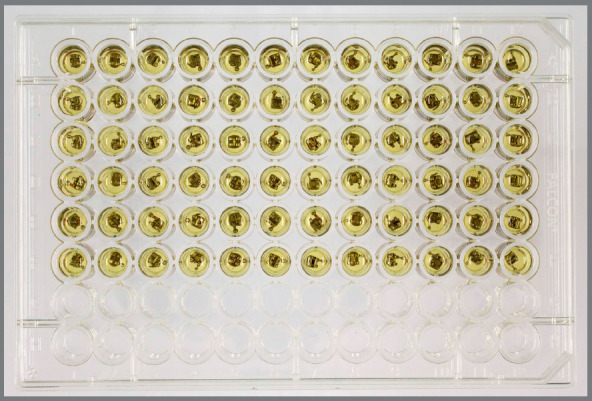
Brackets immersed during experiment.

Into a 96-well plate, the previously sterilized brackets were carefully immersed in saliva for one hour, so that each bracket would occupy a well. After this period, the saliva was removed, the brackets were washed with phosphate buffer solution (PBS), and added to another plate along with 200 µL (in each well) of the bacterial suspension prepared as described in Figure 2. After inoculation, the plate was kept at 37°C, 5% CO_2_ for 72 hours.

Posteriorly, the brackets were removed from the wells and carefully transferred to Eppendorf-type tubes (Eppendorf^®^, Hamburg, Germany) containing 1mL of PBS, which were sonicated for 10 minutes, to separate bacteria adhered to biofilm from the brackets.

To quantify bacterial adherence, serial dilution and plating on BHI agar plates added with sheep’s blood were performed. Colony Forming Units (CFU) counts were performed after 48 hours of bacterial growth on the plates at 37ºC and 5% CO_2_. Thus, the higher the number of CFUs, the greater the number of viable bacteria that adhered to the bracket surface throughout the experiment.

### STATISTICAL ANALYSIS

Data were initially evaluated for their distribution and, after finding non-normal distributions; the groups were compared with the application of the Kruskal-Wallis test. If statistical significance was detected, any differences in pairwise comparisons were verified by applying the Dunn *post-hoc* test. Statistical significance was set at 5% (*p*< 0.05).

## RESULTS

**Figure 3:**
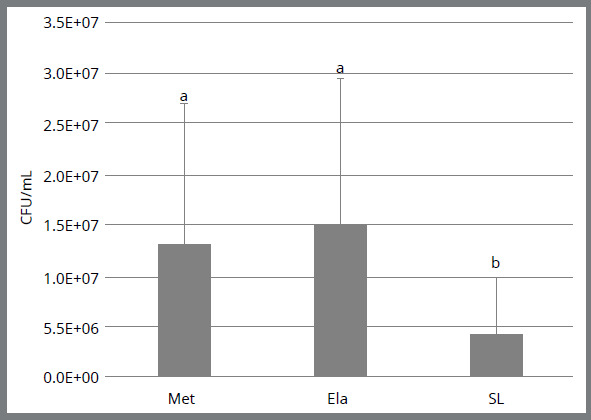
Analysis of biofilm formation by CFU/mL counting in the different types of brackets/ ligatures. Different letters mean statistically significant differences.

Comparative analyzes between types of bracket / ligatures, regardless of the brand are depicted in the Figure 3. Self-ligating brackets (mean: 2.5 x 10^6^; standard deviation: 5.8 x 10^6^; median: 1.9 x 10^6^; quartile 1: 7.0 x 10^5^; quartile 3: 8.5 x 10^6^) presented a significantly lower amounts of CFU/mL (*p*< 0.05), when compared to conventional brackets with metallic (mean: 1.3 x 10^7^; standard-deviation: 1.4 x 10^7^; median: 9.0 x 10^6;^ quartile 1: 3.1 x 10^6^; quartile 3: 1.6 x 10^7^) and elastic (mean: 1.5 x 10^6^; standard-deviation: 1.5 x 10^7^; median: 1.0 x 10^7^; quartile 1: 4.5 x 10^6^; quartile 3: 1.5 x 10^7^) ligatures, which might suggest that, overall, biofilm accumulation in self-ligating brackets is lower.

However, no statistically significant differences were found between the bracket / ligature types in comparisons performed within each commercial brand individually, except for the Abzil^®^ bracket models (Table 2). In the paired comparison between Abz-SL and Abz-Ela, a significantly higher amount of CFU/mL was observed for the latter. In addition, when the Abz-SL and Abz-Met groups were compared, significantly higher amounts of CFU/mL were observed for Abz-Met; but without significant differences when these and the conventional brackets with elastic ligatures (Abz-Ela) were compared with each other (Table 2).

**Table 2:** Descriptive and inferential statistics comparing study groups.

Groups	UFC/mL (median)	UFC/mL (Q1/Q3)	UFC/mL (mean/SD)	Kruskal-Wallis (p-valor)	Dunn (p-valor)	
Abz-Met	7.6x10^6^	2.0x10^5^/	1.7x10^7^/	p = 0.0058	Abz-Met vs. Abz-Ela	p=0.9784
		4.3x10^7^	(2.1x10^7^)			
Abz-Ela	6.0x10^6^	1.1x10^6^/	1.8x10^7^/		Abz-Met vs. Abz-SL	p=0.0063
		4.6x10^7^	(2.3x10^7^)			
Abz-SL	7.0x10^5^	5.5x10^5^/	2.1x10^6^/		Abz-Ela vs. Abz-SL	p=0.0069
		5.1x10^6^	(3.6x10^6^)			
Mor-Met	1.2x10^7^	7.5x10^6^/	1.3x10^7^/	p = 0.6842		
		1.8x10^7^	(5.0x10^6^)			
Mor-Ela	8.8x10^6^	5,0x10^6^/	1.1x10^7^/			
		2.0x10^7^	(8.4x10^6^)			
Mor-SL	7.0x10^6^	1,7x10^6^/	9.4x10^6^/			
		1.9x10^7^	(8.9x10^6^)			
3M-Met	9.0x10^6^	4.6x10^6^/	1.1x10^7^/	p = 0.7720		
		1.9x10^7^	(7.4x10^6^)			
3M-Ela	9.5x10^6^	4.0x10^6^/	1.3x10^7^/			
		2.4x10^7^	(1.1x10^7^)			
3M-SL	2.7x10^6^	9.5x10^5^/	4.3x10^6^/			
		9.2x10^6^	(4.1x10^6^)			
GAC-Met	5.3x10^6^	1.9x10^6^/	1.2x10^7^/	p = 0.7369		
		3.0x10^7^	(1.9x10^7^)			
GAC-Ela	1.4x10^7^	8.0x10^6^/	1.8x10^7^/			
		3.2x10^7^	(1.6x10^7^)			
GAC-SL	1.7x10^6^	3.7x10^5^/	1.4x10^6^/			
		2.0x10^6^	(8.0x10^5^)			

As for the comparisons performed between commercial brands, considering each bracket / ligature type, no significant differences were observed for brackets with metallic (*p*= 0.4852) or elastic (*p*= 0.7120) ligatures. However, among self-ligating brackets, significant differences were observed (*p*= 0.0474), and the GAC^®^ brackets presented relatively higher bacterial adhesion rates. However, when the groups were compared pairwise, this difference only reached statistically significance when GAC-SL was compared to Abz-SL (*p*= 0.0071).

## DISCUSSION

This study aimed at evaluating SM adherence in self-ligating and conventional brackets of different models and ligature types, through the conduction of an *in vitro* experimental design and microbiological analyses. SM strains were used in this study, as this is considered to be the most important microorganism responsible for caries and enamel demineralization.^33^ Furthermore, several studies have already observed that SM levels significantly increase during orthodontic treatment.^34^ ^,^ ^35^

For this study, it was also decided to test commercially relevant brackets, which are usually available for orthodontists. Hence, although this research has been conducted according to a laboratory methodology - and, therefore, with restricted practical applicability -, the preset results can still serve as a useful parameter to help clinicians choosing their material. Thus, *in vitro* studies might be quite relevant, due to the application of rigorous control during the conduction of experiments; and, therefore, they also provide adequate power to evaluate the influences to be potentially exerted by variables, individually. Therefore, since *in vitro* studies are originally conceived to create controlled experimental scenarios, sample calculation may not be considered as mandatory. Still, in order to cover variability, this experiment was performed in triplicate.

One of the results demonstrated that, when bracket / ligature types were compared, without considering the commercial brands, significantly lower bacterial adhesion was observed for the self-ligating brackets. This fact refutes a previous research^36^ that, despite having found differences between bracket models, did not attribute higher degrees of bacterial adhesion to self-ligating brackets. Presumably, such disagreement between results may be associated with the self-ligating bracket commercial brands analyzed in that study,^36^ which differed from those evaluated here.

Thus, it is important to emphasize that any attempt to compare the present results with the ones provided by literature should ideally be made considering the specific bracket models tested here. In this sense, Garcez et al^37^ evaluated GAC^®^ brackets according to microbiological methodology. Unlike the results obtained here, however, those authors^37^ observed that conventional brackets with elastic ligatures adhered significantly more biofilm than self-ligating brackets or brackets with metallic ligatures. Although the present data also indicated a tendency for greater bacterial adhesion for the elastic ligature brackets, this difference was not statistically significant in the analysis of GAC^®^ brackets.

Tupinambá et al^38^ also comparatively evaluated conventional and self-ligating brackets - in this case, from Morelli^®^ commercial brand. While no significant differences were observed by the analysis employed here, the authors of that study^38^ found lower bacterial adhesion to conventional brackets. However, these were processed without the presence of any type of ligature, either metallic or elastic. That might have been one of the reasons why results from both studies are not in accordance. 

By analyzing potential differences between the types of ligatures for each brand individually, differences were statistically significant only for the Abzil^®^ models, with self-ligating having lower biofilm adhesion than the conventional brackets. Whereas one of the major appeals used by self-ligating bracket manufacturers refers to the lower capacity of this type of bracket to accumulate biofilm,^29^ ^,^ ^30^ the data from this study indicated advantages only for Abzil^®^ commercial brand.

Among the four types of self-ligating brackets tested in this study, GAC^®^ showed the highest SM adhesion rates, especially when compared the Abzil^®^ self-ligating brackets. Thus, the most relevant results of this study indicate, on the one hand, the potential superiority of Abzil^®^ self-ligating brackets among the other models from the same brand; and, on the other hand, possible inferiority of the GAC^®^ brackets among the self-ligating bracket models tested in this study.

However, despite possible differences, the results demonstrated here have limited clinical applicability, as already mentioned. Clinical studies still present controversial conclusions regarding the influence of bracket design (conventional *versus* self-ligating) on SM colony formation and adhesion^39^ or upon oral microbiota alteration.^40^ However, according to data collected by a systematic review,^41^ the periodontal status of orthodontic patients seems to remain equally altered, whether by the use of conventional or self-ligating brackets. Such tendency could be noticed, even in a study^42^ evaluating the clinical performance of Abzil^®^ self-ligating brackets, which presented, in this study, the best performance in a laboratorial context.

Thus, based on the data collected in this study, further attempts at controlled clinical studies are encouraged. In addition to including commercially available bracket brands, future studies should also include clinically relevant outcomes, related mainly to the periodontal conditions resulting from the installation of conventional and self-ligated bracket models, and the occurrence of white spot lesions.

## CONCLUSIONS

Self-ligating brackets are likely to present lower rates of biofilm adhesion, particularly Abzil^®^ and GAC^®^ self-ligating brackets. While Abzil^®^ self-ligating brackets are likely to present lower rates of SM biofilm adhesion, when compared to conventional brackets of the same brand (associated with elastic or metallic ligatures), GAC^®^ self-ligating brackets are less likely to accumulate biofilm, especially if compared to Abzil^®^ self-ligating brackets.
